# Data supporting that miR-92a suppresses angiogenic activity of adipose-derived mesenchymal stromal cells by down-regulating hepatocyte growth factor

**DOI:** 10.1016/j.dib.2015.12.021

**Published:** 2015-12-17

**Authors:** Anastassia Efimenko, Georgiy Sagaradze, Zhanna Akopyan, Tatiana Lopatina, Natalia Kalinina

**Affiliations:** Faculty of Medicine, Lomonosov Moscow State University, 31-5, Lomonosovsky av, Moscow 119191 Russia

**Keywords:** Mir-92a, Nucleofection, Cell viability, Capillary-like tube formation

## Abstract

This article contains the full list of miRNAs expressed in cultured mesenchymal stromal cells, which were isolated from human adipose tissue. We provide here data regarding the effect of miR-92a overexpression on MSCs viability and cellular content of HGF and angiopoietin-1. These are followed by the data regarding the effect of conditioned medium of MSC transfected with pre-miR-92a, anti-miR-92a or scramble oligos on HUVEC viability as well as their tube formation efficiency. We also demonstrate here data regarding the effect of extracellular vesicle depletion from MSCs conditioned medium on its ability to stimulate the tube formation by HUVEC. Data interpretation and discussion can be found in Kalinina et al. (2015) [1].

**Specifications Table**

**Table:** 

Subject area	Biology
More specific subject area	Mesenchymal stromal cells and tissue regeneration
Type of data	Table, graphs and microphotographs
How data was acquired	Illumina Human v2 microRNA panel was used to obtain a list of evidently expressed microRNAs. Capillary-like structures were assayed in 24 h under the light microscope (Leica). Concentrations of HGF and angiopoetin-1 in lysates of MSCs transfected with pre-miR-92a (Ambion), anti-miR-92a or pre-miR negative control #1 were measured by ELISA kits (R&D Systems).
Data format	Analyzed
Experimental factors	MSC nucleofected with pre-miR-92a, anti-miR-92a or scramble oligos; MSC conditioned medium applied to HUVEC, recombinant human HGF added
Experimental features	microRNA content in adipose-derived mesenchymal stromal cells was analysed. Adipose-derived MSCs were nucleofected with pre-miR-92a, anti-miR-92a or scramble oligos. HGF and angiopoietin-1 were measured in cell lysates by ELISA. Conditioned medium of transfected cells was applied to HUVEC and tube formation was assessed. Extracellular vesicles were depleted by ultracentrifugation.
Data source location	Lomonosov Moscow State University, Moscow, Russia
Data accessibility	Data with this article

## **Value of the data**

• List of microRNAs expressed in adipose-derived MSCs will help to develop new experiments to study functional activities of these cells.
• Overexpression or down-regulation of miR-92a by nucleofection does not affect MSC viability.
• Our data point to HGF as a new target of miR-92a.

## 1 Data

Using human microRNA v2 panel (Illumina) we have identified 586 miR species, which were evidently expressed in MSCs (see Table 1).

**Fig. 1:**
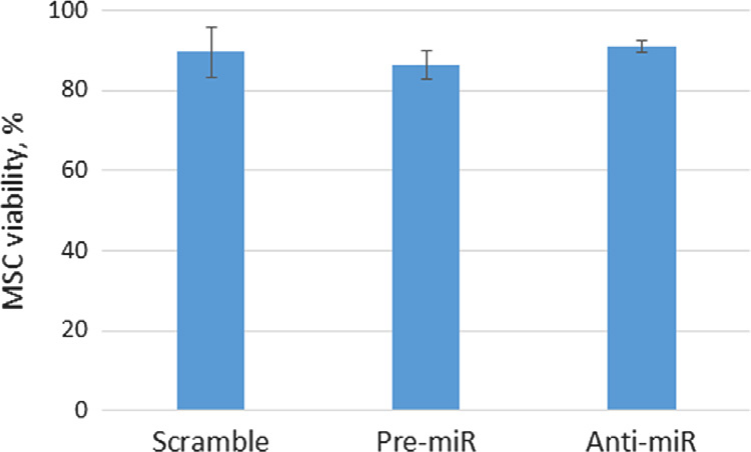
MSC viability. MSC transfected with pre-miR-92a, anti-miR-92a or scramble oilgos were grown for 48 h post-transfection. The portion of viable cells were calculated as a number of Trypan-blue excluding cells per 100 MSCs using Countess Cell Counter, Invitrogen.

We selected miR-92a as a one of the most abundant angio-miRs expressed in MSCs and confirmed its expression by real-time PCR [1]. Then, we overexpressed or down-regulated its content using nucleofection. We examined viability of transfected cells, which was about 90% and did not differ between cells transfected with pre-miR-92a, anti-miR-92a or scramble oilgos (Fig. 1).

**Fig. 2:**
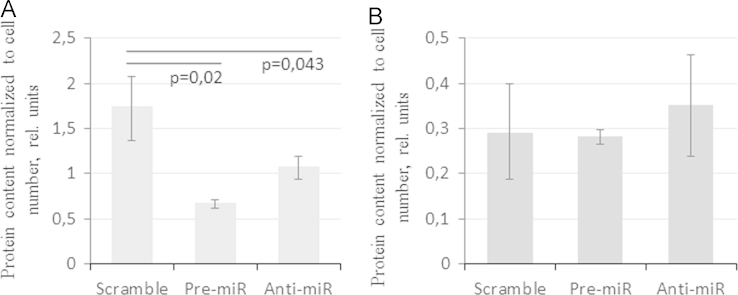
Angiogenic factors content in MSCs 48 h after transfection with either scramble oligos (scramble), pre-miR-92a (pre-miR92a) or anti-miR-92a (anti-miR92a). The content of HGF and angiopoietin-1 in cell lysates was measured by ELISA and normalized to cell counts. Presented are data of 3 experiments. *p<0.05 vs. scramble transfected cells.

We also analyzed the content of HGF and angiopoietin-1 in these cells and found that intracellular content of HGF was 2.6 times lower in MSCs transfected with pre-miR-92a comparing to scramble transfected cells; however, angiopoietin content within MSCs did not change significantly (see Fig. 2).

**Fig. 3:**
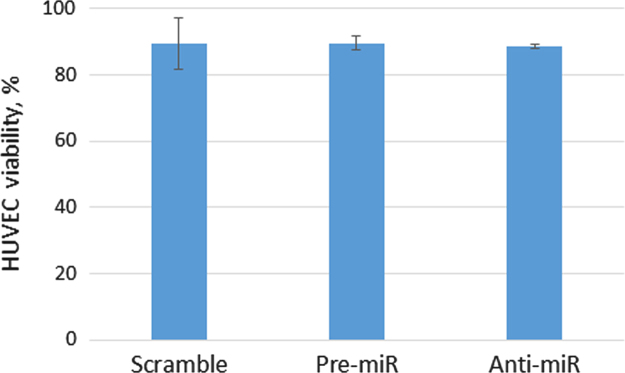
HUVEC viability in the conditioned medium of MSC transfected with pre-miR-92a, anti-miR-92a or scramble oilgos. The portion of viable cells was calculated as a number of Trypan-blue excluding cells per 100 cells.

We collected conditioned medium of MSCs transfected with pre-miR-92a, anti-miR-92a or scramble oilgos, applied it to HUVEC and analyzed their viability. Conditioned medium of transfected MSCs did not affect the viability of HUVEC (see Fig. 3), which was about 90%.

**Fig. 4:**
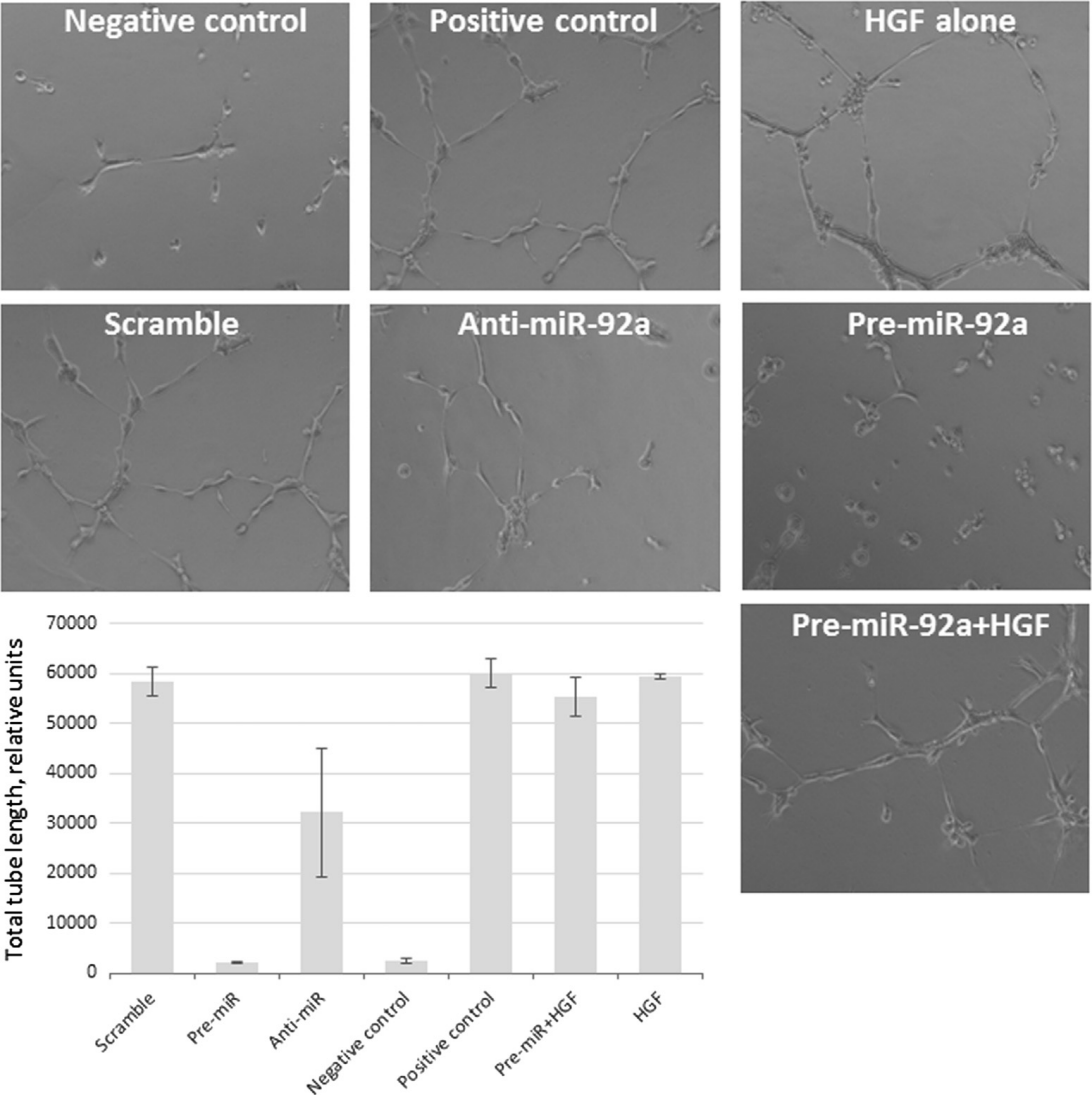
Capillary-like tubules formation in the presence of MSC conditioned medium. Representative microphotographs of capillary-like tubules formed in growth medium without serum (negative control), in the presence of 10% FBS (positive control), in the presence of conditioned medium of MSCs transfected either with scramble oligos (scramble), anti-miR-92a (anti-miR92a), pre-miR-92a (pre-miR92a). Conditioned medium of MSCs overexpressing pre-miR-92a with addition of recombinant human HGF and HGF alone was also used. Graph represents morphometric analysis of capillary-like structures total length per view field. Presented are data of 3 experiments. *p<0.05 vs. conditioned medium of MSCs transfected with pre-miR-92a.

Addition of recombinant HGF but not angiopoietin-1 to the conditioned medium of MSCs transfected with pre-miR-92a restored its ability to stimulate the tube formation by HUVEC (see Fig. 4).

**Fig. 5:**
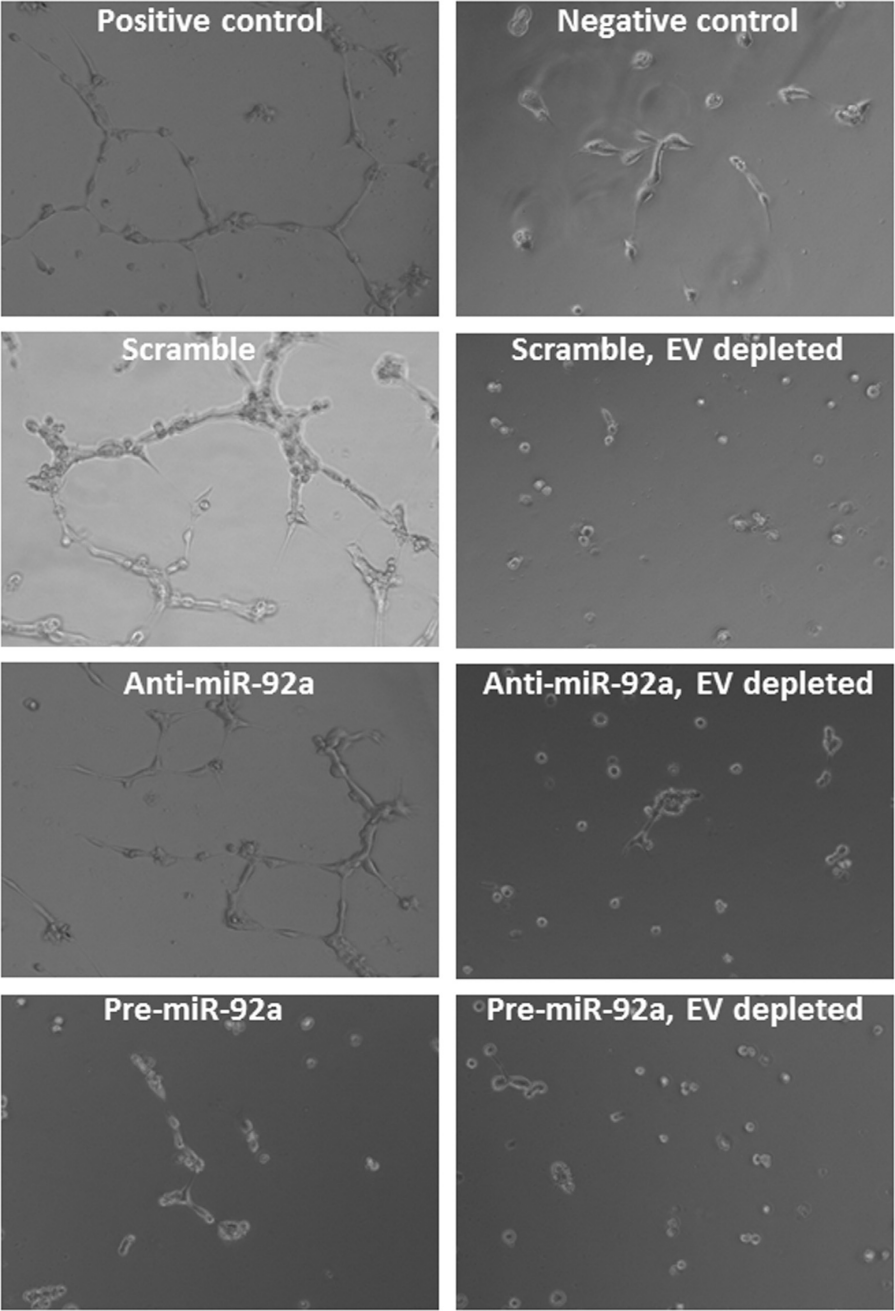
Capillary-like tubules formation in the presence of MSC conditioned medium. Representative microphotographs of capillary-like tubules formed in growth medium without serum (negative control), in the presence of 10% FBS (positive control), in the presence of conditioned medium of MSCs transfected either with scramble oligos (scramble), pre-miR-92a (pre-miR92a) or anti-miR-92a (anti-miR92a) or in the presence of conditioned medium from transfected cells after removing extracellular vesicles by ultracentrifugation.

We also examined if the suppressive effect of conditioned medium of MSCs, which overexpress miR-92a, could be mediated by a direct transfer of this microRNA to endothelial cells by extracellular vesicles. We removed these vesicles from conditioned medium by ultracentrifugation and analyzed the effect of cleared medium on tube formation. Removal of extracellular vesicles completely abrogated the ability of conditioned medium to induce tube formation (see Fig. 5). Data interpretation and discussion can be found in [1].

## 2 Experimental design, materials and methods

### 2.1 Cell culture

MSCs were isolated from subcutaneous fat tissue of healthy young donors using enzymatic digestion as previously described [2]. All donors gave their informed consent and the local ethics committee approved the study protocol. Cells were cultured in AdvanceSTEM Mesenchymal Stem Cell Media containing 10% AdvanceSTEM Supplement (HyClone), 1% antibiotic–antimycotic solution (HyClone) at 37 °C in 5% CO_2_ incubator. Cells were passaged at 70% confluency using HyQTase solution (HyClone). For the experiments, MSCs cultured up to 3rd–4th passages were used.

Human umbilical vein endothelial cells (HUVEC) were isolated from human umbilical cord vein as previously described [3]. Cells were cultured on gelatin-coated plastic in endothelial growth medium (EGM-2, Lonza) and used for experiments at 3–4 passages.

### 2.2 miRNA isolation, hybridization and real-time PCR

Total RNA was extracted from MSCs with Ambion® mirVana™ miRNA Isolation Kit according to manufacture instruction. 200 ng of total RNA was processed according to MicroRNA Assay Guide (Illumina) using Human v2 microRNA panel (1146 probes). Data acquisition and analysis of evidently expressed microRNA were performed by GenomeStudio software (Illumina) using gene expression module. MiRs with detection *p* value<0.05 were considered as evidently expressed. MiR-92a in MSCs was detected using miRVana qRT-PCR miRNA detection kit (Ambion), according to manufacturer׳s protocol. Reverse transcription was performed during 30 min at 37 °C using 25 ng of RNA and hsa-miR-92a RT-Primer (Ambion). Real-time PCR was performed using hsa-miR92a – qRT-PCR assay primer (Ambion) and ready-to-use reaction mix, containing DNA polymerase, SYBR Green and ROX (Evrogen) in 7500 Fast Real-time PCR system (Applied Biosystems).

### 2.3 Cell viability

Viability of MSCs transfected with pre-miR-92a, anti-mi-92a or scramble oligos was assessed 48 h post-transfection. HUVEC viability was analyzed after 24 h incubation in the conditioned medium of MSCs. To assess the viability cells were trypsinized and counted using Trypan blue staining on Countess Automated Cell Counter.

### 2.4 in vitro tube formation assay

HUVEC were seeded in 48-well plates coated with growth factor reduced Matrigel (BD Bioscience, 150 µl per well) in concentration 2×10^4^ cells per well and MSC conditioned media (300 µl per well) were added [4]. Three wells were used for each sample of conditioned medium. Supplement-free serum-free endothelial basal medium (EBM-2, Lonza) was utilized as a negative control; endothelial growth medium (EGM-2, Lonza) with 10% of FBS served as positive control. Plates were placed into CO_2_-incubator at 37 °C and capillary-like structures were assayed in 24 h under the light microscope (Leica). Total length of tubular structures was counted in 5 random fields of view per well (objective 10×) using MetaMorph 5.0 software (Universal Imaging).

To evaluate a significance of HGF and angiopoietin-1 for tube formation we also supplemented the conditioned medium of MSCs overexpressing miR-92a with recombinant growth factors (R&D). To examine an impact of extracellular vesicles on angiogenic action of MSC conditioned medium, we removed them by ultracentrifugation as described in [4].

### 2.5 ELISA of HGF and angiopoietin-1

Concentrations of HGF and angiopoetin-1 in lysates of MSCs transfected with pre-miR-92a (Ambion), anti-miR-92a or pre-miR negative control #1 were measured using ELISA kits (R&D Systems) according to manufacturer instructions.

## Figures and Tables

**Table 1:** List of microRNAs evidently expressed in adipose-derived MSC.

TargetID	Mean	SD
hsa-miR-1280	23567.3	480.0
hsa-miR-21	23136.3	781.4
hsa-let-7a	22779.0	692.8
hsa-miR-214	22123.8	804.3
solexa-3927-221	21213.3	1085.0
hsa-miR-221	20160.3	954.3
hsa-miR-23a	19960.5	719.2
hsa-miR-199a:9.1	19815.5	1912.1
hsa-miR-720	19392.3	513.3
hsa-let-7g	19323.3	974.2
hsa-miR-125b	18808.8	643.9
hsa-miR-24	18341.5	1204.0
HS_22.1	18187.5	961.4
hsa-let-7f	18114.5	609.4
hsa-miR-320d,hsa-miR-320b,hsa-miR-320a	18100.8	428.0
hsa-miR-1274b	17902.8	2536.3
hsa-miR-26a	17701.5	1134.3
hsa-let-7e	17673.3	2166.1
hsa-miR-768-3p:11.0	17642.0	2731.8
hsa-miR-100	17579.3	1745.4
hsa-let-7i	17241.8	804.4
hsa-miR-923	16997.3	3498.3
hsa-let-7b	16807.8	585.4
hsa-miR-222	16276.8	1295.1
hsa-miR-125a-5p	16050.5	1068.0
hsa-miR-199a-3p,hsa-miR-199b-3p	15980.3	1402.7
hsa-miR-92a	15764.3	650.1
hsa-let-7c	15695.5	2136.7
hsa-miR-151-5p	15560.0	1490.1
hsa-miR-23b	15545.5	2236.8
hsa-miR-1308	15318.5	2330.0
hsa-miR-196a	15272.8	645.1
hsa-miR-16	15181.3	1449.2
hsa-miR-25	15137.8	1294.5
hsa-miR-193a-5p	15061.8	978.5
hsa-miR-27a	15018.3	2589.6
solexa-555-1991	14762.5	897.6
hsa-miR-143	14714.8	1900.5
hsa-miR-15b	14409.3	884.1
HS_100	14373.3	1144.9
hsa-let-7d	14286.3	1618.1
hsa-miR-1274a	13864.0	1486.9
hsa-miR-145	13787.3	1564.7
hsa-miR-31	13704.5	1530.4
hsa-miR-574-3p	13679.8	1696.2
hsa-miR-152	13586.8	2963.6
hsa-miR-26b	13480.8	1235.7
hsa-miR-1260	13472.3	1928.7
hsa-miR-1246	13409.8	2074.7
hsa-miR-1826	13393.8	3269.9
HS_192.1	13391.0	2645.2
solexa-2952-306	13256.3	953.8
hsa-miR-424	13215.3	4095.2
hsa-miR-191	13163.8	1851.0
hsa-miR-197	12943.0	1094.8
hsa-miR-98	12607.5	1564.1
hsa-miR-199a-5p	12574.5	3478.1
HS_204.1	12545.0	1184.5
hsa-miR-29a	12474.3	2501.4
HS_263.1	12208.3	2000.0
hsa-miR-224	11944.3	1349.4
hsa-miR-10b	11895.3	1703.7
hsa-miR-27b	11848.5	3310.9
hsa-miR-1201	11518.3	2715.9
hsa-miR-20a	11454.3	2086.0
hsa-miR-30d	11199.5	2248.6
hsa-miR-503	11092.3	1588.7
solexa-3464-254	11059.3	391.3
hsa-miR-132	10687.5	1492.4
hsa-miR-768-5p:11.0	10639.3	1341.5
hsa-miR-10a	10583.8	1824.0
hsa-miR-155	10402.0	2429.4
HS_243.1	10376.3	2551.2
hsa-miR-193b	10359.5	2138.7
hsa-miR-664	10341.0	1762.0
hsa-miR-432	10037.0	2265.1
hsa-miR-195	9968.0	2337.5
hsa-let-7d	9928.8	783.0
hsa-miR-29b-1	9900.3	1879.9
hsa-miR-34a	9880.8	2109.6
hsa-miR-99b	9711.8	2675.7
hsa-miR-30c	9600.3	2441.4
hsa-miR-134	9303.3	934.1
hsa-miR-93	9285.0	592.1
hsa-miR-382	9096.3	1240.3
hsa-miR-92b	8970.5	814.7
hsa-miR-151-3p	8963.0	1436.3
hsa-miR-484	8832.0	1093.8
hsa-miR-28-5p	8679.8	2117.9
hsa-miR-455-3p	8657.8	1939.9
hsa-miR-423-3p	8573.5	1260.9
hsa-miR-424	8397.5	1168.2
solexa-51-13984	8340.0	850.3
hsa-miR-409-3p	8295.8	866.6
HS_284.1	8291.0	1037.4
hsa-miR-374a	8283.5	2769.2
hsa-miR-30e	8194.8	2534.1
hsa-miR-1228	7994.8	492.6
hsa-miR-29b	7992.3	774.7
hsa-miR-148a	7992.0	3801.0
hsa-miR-185	7845.3	1517.6
hsa-miR-15a	7627.3	3472.2
hsa-let-7b	7564,8	881.2
hsa-miR-365	7544.3	1540.2
hsa-miR-493	7387.3	1028.6
hsa-miR-324-5p	7379.0	1880.9
hsa-miR-361-5p	7259.5	2303.2
hsa-miR-544	7162.3	3061.8
hsa-miR-324-3p	7112.3	1953.1
hsa-miR-30b	7072.0	1144.3
hsa-miR-379	6963.3	771.4
hsa-miR-342-3p	6835.3	1553.5
hsa-miR-99a	6792.0	3415.3
hsa-miR-22	6725.0	3846.5
hsa-miR-328	6488.0	1575.3
hsa-miR-151:9.1	6460.3	2527.1
hsa-miR-146a	6456.3	2689.7
solexa-539-2056	6418.3	3114.5
HS_96	6396.5	551.2
hsa-miR-196b	6232.5	647.2
hsa-miR-181b	6187.5	2869.8
solexa-9029-92	6172.0	299.1
hsa-miR-423-5p	6119.0	1363.2
hsa-miR-615-3p	6081.8	332.8
hsa-miR-708	6033.8	2431.3
hsa-miR-411	5970.5	1412.8
hsa-miR-130b	5941.5	1460.8
hsa-miR-146b-5p	5919.0	3049.7
hsa-miR-329	5866.5	1477.3
solexa-8048-104	5741.5	2872.2
hsa-miR-22	5670.8	2076.8
hsa-miR-130a	5667.3	853.1
hsa-miR-181a	5661.5	4037.6
hsa-miR-199b-5p	5543.8	1838.6
hsa-miR-337-3p	5513.0	1579.9
hsa-miR-28-3p	5509.5	1213.1
hsa-miR-140-3p	5425.3	814.1
hsa-miR-1275	5317.8	1528.2
hsa-miR-218	5297.0	488.0
hsa-miR-30a	5277.8	2734.4
hsa-miR-106b	5075.3	1656.1
hsa-miR-425	4907.8	3170.9
hsa-miR-886-5p	4860.3	632.8
hsa-miR-17	4835.8	558.0
solexa-8211-102	4810.8	3885.4
solexa-499-2217	4718.8	514.6
hsa-miR-103	4697.0	1083.3
hsa-miR-574-5p	4654.8	1382.9
hsa-miR-594:9.1	4525.3	1320.1
hsa-miR-939	4440.3	524.5
hsa-miR-889	4371.3	835.2
hsa-miR-877	4359.5	2022.5
hsa-miR-145	4353.5	2399.0
hsa-miR-576-5p	4334.8	688.7
hsa-miR-744	4311.3	520.4
solexa-578-1915	4307.3	785.1
HS_188	4275.3	399.0
hsa-miR-331-3p	4249.8	507.8
hsa-miR-370	4245.3	941.4
hsa-miR-7	4233.0	887.0
hsa-miR-129-5p	4125.3	289.4
hsa-miR-127-3p	4077.5	1046.9
hsa-miR-625	4070.8	855.9
HS_108.1	4007.3	1646.7
HS_29	3980.5	1256.6
hsa-miR-518a-3p	3974.5	439.7
hsa-miR-542-3p	3970.3	2890.8
hsa-miR-1249	3959.0	1498.5
hsa-miR-106a	3835.0	825.1
hsa-miR-193b	3768.3	1340.9
hsa-miR-24-2	3718.0	1956.8
hsa-miR-532-3p	3716.8	1867.2
hsa-miR-128	3681.0	888.9
hsa-miR-628-5p	3673.0	1407.6
hsa-miR-29c	3658.8	2366.8
hsa-miR-663	3592.3	588.6
hsa-miR-625	3586.5	745.3
hsa-miR-204	3579.8	443.0
hsa-miR-30c-1	3551.8	1099.1
hsa-miR-376c	3492.5	589.6
hsa-miR-221	3461.0	2028.3
hsa-miR-539	3454.3	466.6
hsa-miR-30a	3437.3	1699.3
hsa-miR-548b-5p	3402.5	343.6
hsa-miR-886-3p	3352.5	649.1
hsa-miR-7-1	3338.0	3726.7
hsa-miR-1300	3277.5	2082.2
hsa-miR-450a	3205.5	2247.8
hsa-miR-628-3p	3185.8	1065.8
hsa-miR-15b	3160.3	764.0
hsa-miR-130b	3152.0	1059.8
hsa-miR-372	3144.3	302.8
hsa-miR-433	3129.8	378.9
hsa-miR-335	3084.5	1863.3
hsa-miR-126	3052.3	703.7
hsa-miR-675	3024.3	591.5
hsa-miR-487b	3016.0	534.6
hsa-miR-16-2	3011.0	1345.6
hsa-miR-126	2964.3	2488.8
hsa-miR-299-5p	2726.3	738.4
hsa-miR-30e	2707.3	1939.7
hsa-miR-10a	2704.0	604.5
hsa-miR-31	2686.3	2011.9
hsa-miR-517a	2681.8	439.7
solexa-4793-177	2568.5	298.2
hsa-miR-421	2521.0	728.3
hsa-miR-342-5p	2514.5	439.7
hsa-miR-654-5p	2511.8	472.9
hsa-miR-940	2479.5	212.6
HS_239	2468.8	749.4
hsa-miR-143	2447.8	213.5
hsa-miR-138	2432.8	1123.8
hsa-miR-485-3p	2414.8	1197.9
hsa-miR-450b-5p	2403.8	1373.2
hsa-miR-137	2398.5	2711.8
HS_244	2385.8	156.3
hsa-miR-378	2350.5	611.8
HS_275	2310.3	174.3
hsa-miR-500	2307.8	1487.3
hsa-miR-499-5p	2305.3	367.1
hsa-miR-148b	2279.5	2879.8
hsa-miR-664	2117.5	660.3
hsa-miR-301a	2088.8	190.6
hsa-miR-214	2069.5	1221.0
hsa-miR-181a-2	2052.5	1471.7
hsa-miR-335	1995.0	1939.3
HS_38.1	1993.3	175.6
hsa-miR-29a	1978.5	1742.2
hsa-miR-362-3p	1967.5	421.5
hsa-miR-23a	1926.3	661.7
HS_152	1902.3	74.4
HS_303_a	1883.8	254.7
hsa-miR-548d-5p	1872.8	480.8
hsa-miR-339-5p	1872.3	419.9
hsa-miR-502-3p,hsa-miR-500	1848.5	321.7
hsa-miR-560:9.1	1840.8	249.8
hsa-miR-189:9.1	1812.8	638.2
hsa-miR-125b-1	1798.3	557.2
hsa-miR-585	1794.8	360.7
hsa-miR-210	1787.5	616.7
hsa-miR-494	1746.5	766.3
hsa-miR-769-5p	1733.3	264.7
hsa-miR-532-5p	1717.5	465.2
HS_94	1704.8	219.0
hsa-miR-493	1696.3	786.1
hsa-miR-517c	1695.5	633.4
hsa-miR-565:9.1	1675.3	493.9
hsa-miR-454	1663.3	527.2
hsa-miR-323-3p	1660.8	616.3
solexa-9655-85	1649.5	120.6
hsa-miR-92b	1644.3	727.9
hsa-miR-1287	1643.0	272.5
hsa-miR-212	1632.3	810.2
HS_199	1621.5	211.5
hsa-miR-1307	1608.3	381.4
hsa-miR-154	1606.0	957.6
hsa-miR-1301	1584.5	285.1
hsa-miR-1284	1539.0	114.2
hsa-miR-485-5p	1535.8	381.7
hsa-miR-194	1534.8	88.7
hsa-miR-624	1526.8	622.9
hsa-miR-519b-3p	1509.8	95.1
HS_113	1492.0	41.2
HS_71.1	1466.5	89.0
hsa-miR-216b	1444.8	389.1
hsa-miR-1285	1442.0	443.3
HS_150	1428.5	143.1
hsa-miR-27b	1396.0	892.5
HS_268	1390.5	100.5
hsa-miR-133a	1384.5	203.6
HS_156	1361.5	984.0
HS_139	1354.8	105.8
HS_19	1354.0	97.7
hsa-miR-551a	1344.8	195.1
hsa-miR-505	1342.5	835.9
HS_184	1299.3	63.8
hsa-miR-1271	1277.0	806.7
hsa-miR-491-5p	1243.0	206.9
HS_114	1242.8	196.3
hsa-miR-381	1231.0	278.1
hsa-miR-627	1210.3	356.3
hsa-miR-181a	1200.3	46.4
hsa-miR-660	1192.0	1533.2
hsa-miR-1224-3p	1182.8	115.5
hsa-miR-449b	1178.3	39.1
hsa-miR-140-5p	1161.0	840.4
hsa-miR-1228	1158.3	214.7
hsa-miR-501-3p	1156.0	167.3
hsa-miR-548a-3p	1153.3	66.9
hsa-miR-1299	1153.0	185.4
hsa-miR-296-3p	1151.8	410.7
HS_32	1139.5	123.6
hsa-miR-1254	1130.5	125.3
**hsa-miR-200c**	**1126**.**8**	**208.4**
HS_81	1122.8	90.8
hsa-miR-671:9.1	1109.5	455.8
hsa-miR-34c-3p	1103.8	866.9
hsa-miR-1234	1097.5	302.5
hsa-miR-34b	1097.3	369.2
hsa-miR-125a-3p	1073.3	88.8
hsa-miR-1296	1071.8	354.0
hsa-let-7e	1068.8	429.2
hsa-miR-571	1065.8	110.0
hsa-miR-149	1061.5	533.1
HS_166.1	1056.8	305.9
hsa-miR-19b	1041.0	80.3
hsa-miR-298	1038.0	120.1
hsa-miR-1233	1037.5	183.0
hsa-let-7f-1	1036.5	333.0
hsa-miR-362-5p	1034.8	270.1
hsa-miR-99b	1034.5	449.7
hsa-miR-105	1021.0	259.3
hsa-miR-23b	999.8	338.4
hsa-miR-409-5p	997.5	728.5
hsa-miR-629	988.5	336.3
hsa-miR-369-5p	986.8	304.0
hsa-miR-602	983.8	75.8
hsa-miR-875-5p	982.3	130.0
hsa-miR-495	974.8	217.1
hsa-miR-584	969.3	310.9
hsa-miR-18a	957.5	786.4
hsa-miR-501-5p	957.3	246.6
hsa-miR-187	955.5	126.8
hsa-miR-1257	948.5	41.4
hsa-miR-25	947.5	141.3
hsa-miR-452:9.1	946.0	343.0
hsa-miR-1267	940.0	146.5
hsa-miR-505	926.8	575.6
hsa-miR-642	919.0	295.5
HS_10	918.5	780.9
hsa-miR-18a	916.8	353.2
hsa-miR-615-5p	912.5	284.8
hsa-miR-517	908.5	116.3
hsa-miR-26b	904.0	564.8
hsa-miR-890	896.5	71.4
hsa-miR-452	892.3	397.7
hsa-miR-431	889.5	179.5
hsa-miR-186	874.8	415.7
HS_303_b	863.8	241.4
hsa-miR-671-3p	862.3	430.5
hsa-miR-192	861.8	991.3
hsa-miR-609	859.8	29.5
hsa-miR-194	859.3	1047.4
HS_97	846.8	133.1
hsa-miR-369-3p	845.0	191.3
hsa-miR-942	844.5	187.6
HS_116	842.3	321.3
hsa-miR-34c-5p	831.8	186.6
hsa-miR-603	824.5	574.6
hsa-miR-27a	816.8	557.3
hsa-miR-766	809.5	249.5
HS_85.1	806.3	108.5
HS_260	797.8	173.7
hsa-miR-29c	797.8	204.0
hsa-miR-223	796.0	418.9
hsa-miR-106b	790.0	334.5
hsa-miR-550	783.3	208.7
hsa-miR-30d	781.8	93.6
hsa-miR-195	781.3	98.8
hsa-miR-654-3p	771.8	204.8
hsa-miR-665	762.8	156.5
HS_208	757.5	72.5
hsa-miR-138-1	757.3	398.7
hsa-miR-302d	750.3	319.6
solexa-15-44487	749.5	58.3
hsa-miR-554	738.8	82.8
solexa-9081-91	734.5	42.2
hsa-miR-1204	720.5	64.3
hsa-miR-193a-3p	718.0	357.6
hsa-miR-549	713.8	393.5
hsa-miR-507	711.5	58.1
hsa-miR-497	710.3	339.4
HS_110	706.0	133.1
hsa-miR-361-3p	703.5	321.3
hsa-miR-663b	700.3	68.3
HS_250	697.8	120.2
hsa-miR-483-3p	694.8	312.6
hsa-miR-486-5p	685.0	276.9
hsa-miR-1305	684.8	58.5
hsa-miR-425	682.5	305.4
hsa-miR-545	677.8	116.9
hsa-miR-491-3p	675.8	62.8
HS_52	670.8	54.0
hsa-miR-573	658.5	20.5
hsa-miR-34a	654.8	305.1
hsa-miR-1286	651.3	43.5
HS_209.1	644.3	291.1
hsa-miR-598	643.8	240.9
hsa-miR-371-5p	643.3	68.0
hsa-miR-641	635.0	14.9
hsa-miR-525-3p	634.5	123.4
hsa-miR-655	633.5	296.8
hsa-miR-801:9.1	631.8	58.4
HS_78	626.8	69.8
hsa-miR-33a	625.3	43.6
hsa-miR-16-1	620.8	296.5
hsa-miR-652	619.0	213.5
hsa-miR-411	616.8	79.5
hsa-miR-1245	614.3	280.4
hsa-miR-302b	612.5	278.1
hsa-miR-518c	611.3	38.9
hsa-miR-1263	611.0	39.9
HS_262.1	606.0	33.8
hsa-miR-516a-3p,hsa-miR-516b	600.5	85.7
hsa-miR-513a-5p	598.5	29.9
hsa-miR-502-5p	594.5	192.1
hsa-miR-302b	590.5	107.0
hsa-miR-518d-3p	578.3	59.0
solexa-7534-111	577.5	222.6
hsa-miR-583	576.8	100.9
HS_24	573.5	315.9
hsa-miR-376a	573,5	183.2
hsa-miR-136	572.5	530.1
HS_20	571.3	46.5
hsa-miR-346	570.5	183.7
hsa-miR-576-3p	568.8	221.2
solexa-7509-112	564.0	40.9
HS_279_a	555.8	50.5
hsa-miR-659	548.5	83.4
hsa-miR-188-5p	546.5	120.0
solexa-9578-86	545.5	223.2
hsa-miR-376a:9.1	540.3	236.5
HS_186	536.8	60.1
hsa-miR-379	535.0	218.6
hsa-miR-1225-3p	534.8	118.3
HS_305_b	526.5	115.1
hsa-miR-26a-2	525.3	337.8
solexa-1460-671	519.8	182.3
hsa-miR-525-5p	516.3	24.6
solexa-9124-90	509.0	124.0
hsa-miR-376a	504.8	315.1
hsa-miR-1180	501.8	55.2
hsa-miR-668	500.5	89.6
hsa-miR-101	500.3	103.7
hsa-miR-330-3p	500.0	130.0
HS_160	495.0	74.7
hsa-miR-542-5p	490.5	43.0
hsa-miR-518a-5p,hsa-miR-527	487.5	7.3
solexa-826-1288	487.3	120.0
hsa-miR-99a	487.0	38.7
HS_17	486.3	259.7
hsa-miR-1184	485.5	153.5
hsa-miR-520d-5p	479.0	34.3
hsa-miR-550	478.5	101.5
HS_287	473.5	119.3
hsa-let-7c	468.5	189.5
hsa-miR-374a	465.5	45.5
hsa-miR-651	462.0	49.0
solexa-8926-93	461.3	293.0
**hsa-miR-200b**	**456.8**	**100.3**
hsa-miR-17	449.5	78.6
hsa-miR-19b-2	448.0	17.3
HS_252.1	444.8	12.3
hsa-miR-218-1	444.8	38.8
hsa-miR-616	437.5	247.5
hsa-miR-1226	434.5	133.6
hsa-miR-1225-5p	429.8	321.2
hsa-miR-21	428.3	357.3
hsa-miR-376b	423.0	55.8
hsa-miR-122	421.5	33.6
hsa-miR-30c-2	421.0	134.6
hsa-miR-647	418.8	69.6
hsa-miR-132	418.5	288.2
hsa-miR-367	416.3	17.6
HS_276.1	412.8	50.3
solexa-3022-299	409.0	26.0
hsa-miR-941	408.5	154.1
hsa-miR-299-3p	407.8	54.0
HS_60	406.5	89.3
hsa-miR-591	406.5	28.0
hsa-miR-1197	404.5	91.6
hsa-miR-340	403.8	242.4
HS_142.1	402.0	82.7
hsa-miR-139-5p	399.3	257.8
solexa-3126-285	398.8	184.2
HS_145.1	395.5	30.6
hsa-miR-1294	395.0	95.9
hsa-miR-30b	395.0	129.0
HS_105	394.3	44.1
hsa-miR-1268	393.0	68.3
hsa-miR-302c	392.8	15.3
HS_203	384.8	25.9
hsa-miR-629	384.5	213.8
hsa-miR-518e:9.1	384.3	29.2
HS_126	383.5	34.6
hsa-miR-410	382.5	217.5
HS_149	380.8	167.1
hsa-miR-545:9.1	378.8	140.1
HS_48.1	377.8	65.3
hsa-miR-1207-5p	376.3	32.7
hsa-miR-516a-5p	372.8	60.1
hsa-miR-377	369.0	77.0
HS_196.1	368.5	117.1
hsa-miR-182	368.0	92.5
hsa-miR-452	367.8	29.0
HS_27	363.5	15.6
hsa-miR-564	360.8	60.5
HS_219	351.8	63.6
HS_285	350.3	87.8
hsa-miR-1322	348.8	81.7
hsa-miR-1206	348.0	25.7
hsa-miR-1243	347.0	72.4
hsa-miR-614	345.3	39.1
HS_25	34.8	70.5
hsa-miR-92a-1	340.8	119.4
hsa-miR-296-5p	337.8	54.2
hsa-miR-1248	332.5	86.2
hsa-miR-144	331.5	23.9
HS_6	328.3	74.8
hsa-miR-128a:9.1	326.3	122.1
solexa-5874-144	326.3	35.0
hsa-miR-567	324.0	19.4
hsa-miR-202:9.1	323.3	131.5
hsa-miR-220b	323.0	101.0
HS_90	319.3	26.8
hsa-miR-653	316.3	14.3
hsa-let-7a	313.0	162.2
hsa-miR-107	310.8	109.9
HS_99.1	309.3	51.0
HS_201	307.5	105.4
hsa-miR-767-5p	306.5	38.6
hsa-miR-34b	305.0	43.5
hsa-miR-222	304.8	34.2
hsa-miR-935	303.8	45.1
HS_2	303.5	65.8
HS_122.1	299.5	16.6
hsa-miR-454	297.3	86.9
hsa-miR-181c	296.3	53.6
hsa-miR-380	294.0	38.9
hsa-miR-326	293.5	84.4
hsa-miR-339-3p	293.0	72.7
hsa-miR-338-5p	291.0	28.2
hsa-miR-100	285.8	63.8
hsa-miR-1231	283.5	33.4
HS_138	279.5	21.4
hsa-miR-887	278.3	36.9
hsa-miR-1273	276.0	127.5
hsa-miR-129-3p	275.5	107.2
hsa-miR-1237	274.0	116.0
HS_119	271.0	25.6
hsa-miR-646	268.5	109.8
HS_40	261.8	33.6
hsa-miR-1304	256.0	79.5
hsa-miR-384	255.5	17.2
hsa-miR-371-3p	251.8	143.6
hsa-miR-377	250.5	44.9
hsa-miR-760	250.5	47.3
hsa-miR-455-5p	249.0	129.0
hsa-miR-147b	248.3	21.7
hsa-miR-1323	248.0	22.9
hsa-miR-219-2-3p	246.8	24.5
hsa-miR-596	246.3	42.0
hsa-miR-581	242.0	28.9
hsa-miR-519c-3p	237.3	30.7
hsa-miR-9	234.3	46.0
HS_153	232.8	11.0
hsa-miR-1272	232.5	24.2
hsa-miR-769-3p	230.3	140.8
hsa-miR-643	227.3	25.3
hsa-miR-520a-3p	222.3	30.6
HS_23	221.8	25.9
hsa-miR-323-5p	221.8	55.4
hsa-miR-559	221.0	13.5
hsa-miR-345:9.1	220.8	110.5
hsa-miR-1297	220.5	15.5
hsa-miR-412	218.3	13.7
hsa-miR-624	218.3	18.0
hsa-miR-548c-5p	210.3	36.5
hsa-miR-563	210.3	34.9
hsa-miR-876-3p	208.0	29.9
hsa-miR-135b	207.8	42.0
HS_200	201.3	28.0
hsa-miR-1208	198.0	33.8
hsa-miR-383	196.8	80,2
hsa-miR-453	196.0	23.8
HS_141	195.0	25.1
hsa-miR-331-5p	193.8	11.0
HS_33	193.3	18.0
hsa-miR-548f	189.3	25.2
hsa-miR-206	188.8	17.0
hsa-miR-190	180.8	5.6
hsa-miR-548m	177.5	21.3
hsa-miR-592	171.8	14.8
hsa-miR-1262	159.0	13.3
